# The acceptability of a novel procedure service run by PAs and NPs

**DOI:** 10.1097/01.JAA.0000794988.39630.38

**Published:** 2021-11-23

**Authors:** Nancy Kim, Tara Herbert, Sheyla Marranca, Eric Bergman, Ronald Castillo, Lindsey Romano, Daniel Heacock, William Cushing

**Affiliations:** **Nancy Kim** is an assistant clinical professor of internal medicine at Yale University School of Medicine and Physician Partner, Care Signature, Yale-New Haven Health System in New Haven, Conn. **Tara Herbert, Sheyla Marranca, Eric Bergman, Ronald Castillo, Lindsey Romano,** and **Daniel Heacock** practice in the New England Medical Group Hospitalist Service at Yale-New Haven Hospital. **William Cushing** is executive director of hospital medicine at Yale-New Haven Hospital. The authors have disclosed no potential conflicts of interest, financial or otherwise.

**Keywords:** procedure service, bedside, venous access, PA, NP, hospitalist

## Abstract

**Background:**

Hospitalist physicians are performing fewer procedures because of multiple reasons, including expanded responsibilities beyond their patient panel. A procedure service that offloads hospitalists could expedite these necessary services. An opportunity exists for physician assistants (PAs) and NPs to fill this gap.

**Objective:**

To describe the implementation of a PA- and NP-run procedure service at a large academic hospital.

**Methods:**

This is a retrospective cohort study of procedures by the procedure service at one institution from 2015 to 2019.

**Results:**

Over 5 years, 7,002 procedures were performed, with requests increasing over time. The most frequent procedures were venous access, lumbar puncture, paracentesis, and placement of nasogastric or nasojejunal tubes. Requesting services included hospitalists and residents from internal medicine, surgery, and neurology.

**Conclusions:**

A PA- and NP-run procedure service is well accepted at a large academic hospital despite the lack of involvement by attending physicians. Future directions are focused on augmenting coverage and procedures offered.

Although bedside procedures are considered core competencies by the Society of Hospitalist Medicine, they are performed infrequently by hospitalist physicians.^1^,^2^ Several factors are contributing to this observation. Residency programs no longer require manual training in procedures such as thoracenteses, paracenteses, lumbar punctures, establishing peripheral access, and placing central venous catheters and nasogastric tubes.^3^ This leaves many new attending physicians without technical abilities in these interventions.^4^-^7^ Additionally, hospitalist attending physicians have growing responsibilities, such as rounding on increasingly complex patients, providing rapid response for decompensating patients, consulting on cases for other services, and admitting new patients. Little time remains for hospitalist physicians to perform the number of procedures required to maintain comfort in these skills.^8^

A procedure service offers a potential solution for these necessary medical interventions. This dedicated team of clinicians perform a specified menu of procedures on a daily basis, expediting necessary interventions and improving patient safety and efficiencies in patient throughput.^9^ The team performs procedures at the patient's bedside rather than transferring the patient to a separate procedure suite, which involves transport, wait times, and the introduction of a new consult service and clinicians unfamiliar to the patient. Procedure services are not new but must be adapted to their surroundings to function optimally and meet local needs.^10^ However, standard features of such a service are not clear from the existing literature.

The shift in residency education and the demands on hospitalist physicians have created an opportunity for PAs and NPs. At our institution, PAs and NPs are protected from the expanded roles of responding to rapid response calls, holding the admission pager, and admitting new patients. They have fewer competing priorities for time than the attending physicians, making them ideal candidates to fill this procedure gap. PAs and NPs routinely perform procedures in surgical specialties, emergency medicine, and interventional radiology.^11^ With training, hospitalist PAs and NPs can develop competency in bedside procedures. This creates another avenue for PAs and NPs to function independently while providing an invaluable service to patients and the hospital.

We sought to add to a burgeoning body of knowledge by describing our experience initiating such a service at our institution, a large teaching hospital in the Northeast. Over time, our procedure service has grown and become staffed solely by PAs and NPs (Figure 1). To our knowledge, our procedure team is unique in this regard. We outline the structure of our procedure team, its evolution over time, use over 5 years, and future directions. This model may be replicable in other large hospitals.

**FIGURE 1.**
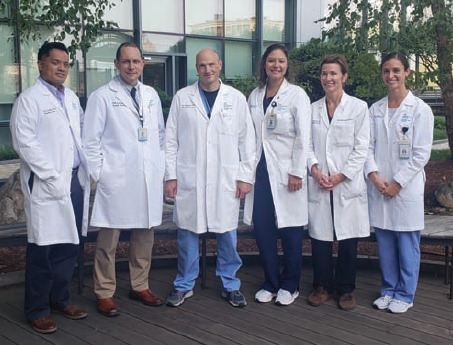
The Yale-New Haven Hospital procedure service team, from left: Ronald Castillo, APRN; William Cushing, PA-C; Eric Bergman, PA-C; Sheyla Marranca, PA-C; Tara Herbert, PA-C; and Lindsey Romano, PA-C

## METHODS

This was a retrospective cohort study of all procedures performed by the procedure service at one institution from January 2015 through September 2019. The study met the definition for clinical quality improvement, so did not require institutional review board review or approval at our institution.

Our institution is licensed for 1,541 beds across two major campuses in the state. We are a tertiary medical center in New England and the primary teaching hospital for our university. As such, we host full residencies for physicians in core areas including internal medicine, surgery, surgical subspecialties, emergency medicine, obstetrics/gynecology, pediatrics, combined internal medicine/pediatrics, and psychiatry.

Before the implementation of the procedure service, procedures such as difficult central venous access placements were performed by the primary team (hospitalist or trainees), interventional radiology, or surgical trainees. The pilot procedure service was established on July 27, 2012, and staffed by one PA and one attending physician. As the number of requests increased, more PAs and NPs were recruited. As the number of PAs and NPs managing the procedure requests grew, the attending physician's involvement was discontinued as he assumed increasing administrative responsibilities for the hospitalist group. Since 2014, the procedure service has been staffed wholly by select hospitalist PAs and NPs who are experts at common inpatient bedside procedures. These experts demonstrated competency in performing these procedures for more than 3 years and were identified to train PAs and NPs as well as residents. In 2019, the procedure service staff consisted of five PAs and one NP. As the team expanded, a formal mechanism to record procedures began in January 2015. Complete data exist through September 2019.

Although no consensus exists on credentialing for bedside procedures, best practices from the literature include the use of competency-based methods such as mastery learning and deliberate practice as effective teaching strategies.^12^,^13^ In that context, our PAs and NPs are trained in an apprenticeship model. Specifically, initial training for hospitalist PAs and NPs includes didactic training and 1:1 support from other experienced members of the procedure service. Additional oversight of the PAs and NPs includes the same institutional focused professional practice evaluations (FPPE) and ongoing professional practice evaluations (OPPE) as physicians. Once they have completed the initial evaluation of competence in the FPPE, PAs and NPs must document annually in the OPPE. Our institution tracks compliance with these performance data via reports generated directly from our electronic medical record (EMR). Lastly, any quality and safety events are reported through the hospital event reporting system and would be referred to our serious event reporting committee.

The procedure service performs procedures independently and/or supervises residents who are not credentialed. They cover the general units (medical, surgical, and neurologic), as well as the ICUs (medical, surgical, and neurologic). One PA or NP is assigned to cover procedures each day. This clinician also provides care for a limited panel of patients on the hospitalist service, as the first point of contact.

In our institution, dedicated teams for thoracenteses and peripherally inserted central catheters (PICCs) are well integrated into the daily work flow. Thus, our procedure service is focused on the following bedside procedures: lumbar punctures, paracenteses, obtaining peripheral venous access, and placing central venous catheters and nasogastric tubes. The procedure service is available from 7 a.m. to 6 p.m. weekdays and 7 a.m. to 5 p.m. weekends. All procedures are done using best practices. A portable ultrasound device is available to the procedure service at all times. Ultrasound guidance is used for all paracenteses and central venous access placements in accordance with the recommendations from the Society of Hospital Medicine Point of Care Ultrasound Task Force.^12^,^14^ Ultrasound is also used for peripheral IVs placed by the procedure service.

### Data collection

All procedures are logged into a central internal database outside of the EMR. Data elements recorded internally include requesting service, procedure requested, time of procedure, and complications. These data are routinely captured in the course of clinical care. Over time, the mechanism for consulting the procedure service changed. In 2012, requests for procedures were made via a unique pager number using a traditional beeper system. As of 2019, consult requests require two steps. First, the requesting clinician directly calls or texts the procedure PA or NP via a secured communication platform (Mobile Heartbeat). This provides an opportunity for clinicians to discuss several important topics, including the indication for and choice of procedure (for example, central venous access rather than peripheral access), consent, and logistical items such as equipment or kits at the bedside. At that time, the procedure service also can determine whether it will perform the procedure or supervise the primary team. The procedure service also reviews any imaging, relevant laboratory tests, and medications for contraindications.

Second, the PA or NP places an order into the EMR after the case is reviewed. A standardized procedure note is then placed in the EMR. Because of these changes in consulting the procedure service and recording notes in the EMR, reliable data can only be reported from 2015 through 2019.

## RESULTS

**FIGURE 2.**
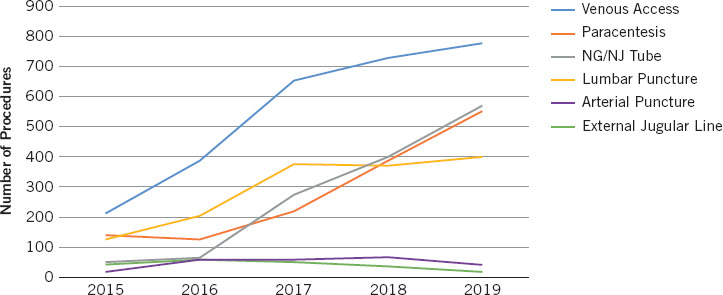
Procedures performed by type from January 2015-December 2019

**FIGURE 3.**
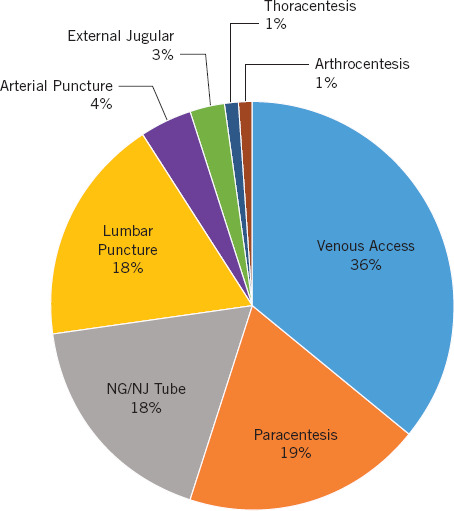
Percentage of procedures performed from January 2015-December 2019

Over the past 5 years, the procedure service has been available to all services in the hospital. From January 2015 to September 2019, a total of 7,002 procedures were performed. The absolute number of procedure requests increased over time (Figure 2). The most common procedures requested in order were venous access (n = 2,818), lumbar puncture (n = 1,390), paracentesis (n = 1,283), and nasogastric/nasojejunal tubes (n = 1,239) (Figure 3). Requesting services varied, with procedures being requested by attending hospitalists as well as residents from internal medicine, surgery, and neurology. All procedures were completed within 24 hours of request. Complications occurred in fewer than 1% of procedures.

## DISCUSSION

The procedure service was a pilot project aimed at providing safe, timely bedside procedures to adult inpatients at a large urban academic institution. The procedure service has become well established in the broader hospital framework and remains well used, with 159 to 220 procedures performed per month (unpublished 2020 data). This far exceeds the number performed per month by residents without access to a procedure service (n = 4.3 to 64.4) reported in the literature.^15^ In the 7 years since the procedure service's inception, its structure has changed from a physician-led team to a PA- and NP-run service. The attending physician who was initially involved was deployed for other roles, given the ability of the PAs and NPs to manage procedures without direct supervision. That said, attending physician involvement is not prohibited, but none of our hospitalist attending physicians has joined the procedure service, presumably because of their many other roles and the fact that procedures are time-consuming. Therefore, using PAs and NPs in this role may become increasingly relevant to other institutions, as some, despite finding the procedure service beneficial, have discontinued their service because of physician faculty attrition.^16^ The emerging role of PAs and NPs on the procedure service is consistent with other literature that demonstrates that these clinicians have an increasing presence in nonvascular invasive procedures.^17^

Overall, the procedure service has had very low rates of complications. Our rates were lower than those reported by others, which found a pooled complication rate of 2.1%; however, our procedures include peripheral access and nasogastric tubes, which tend to be less invasive than the procedures included in the broader literature.^15^ Other literature has also demonstrated that PAs and NPs have the same complication rates as physicians when performing procedures.^18^

Over time, an increasing number of procedures have been requested by a large breadth of services. We were surprised by requests made in the ICU and step-down units, which are closed and have limited censuses. These units, which provide higher levels of care, are staffed with pulmonary fellows and intensivists who presumably are experienced in bedside procedures. The requests coming from surgical subspecialties also were unexpected, given their experience with invasive procedures. This may reflect the increasing daily demands on primary teams because of the complexity of patient case-mix at our institution.

## FUTURE DIRECTIONS

Although a full cost analysis is beyond the scope of this paper, previous work has demonstrated that bedside procedures are cheaper than procedures performed by interventional radiology.^19^ Several features of our procedure service may render it cost-effective for hospitals. First, the service is staffed by PAs and NPs. This way, hospitalist attending physicians can be fully used for patient care. Second, PAs and NPs on the procedure service continue to provide primary coverage for hospitalist patients, and are not subsidized by the hospital as a separate entity. Third, the service provides same-day procedures in almost 100% of cases requested, likely promoting a shorter patient length of stay. This is in line with other literature that states bedside procedures are faster than the same procedure completed by radiology.^20^ This is likely due to the fact that patients are not reliant on the interventional radiology schedule, which is limited by a fixed number of procedure suites.

Historically, our procedure service recoups 10% of its billing for reasons related to contractual fees negotiated between our institution and the insurance companies. For this reason, our procedure service does not routinely bill. However, a recent study found that procedure services are not only cost-effective but can generate revenue for their departments.^21^ Given the financial losses suffered by most hospitals during the COVID-19 pandemic, we are reexploring the profitability of our procedure service, because it could potentially sway hospital leadership to expand the program.^22^

A procedure service also can potentially improve patient satisfaction and safety, two important quality metrics reported nationally.^23^ The service provides a great benefit to patients because it comes to the bedside, eliminating the need for a physical transition to an interventional radiology suite. This reduces disruption and builds efficiency into patients' hospital care. At our institution, the wait times for interventional radiology are a profound source of frustration for patients and clinicians. From a safety perspective, increasing the number of PICCs reduces the need for central venous catheters, reducing the number of potential central line-associated bloodstream infections, a hospital-acquired condition measured by the Centers for Medicare and Medicaid Services (CMS).^24^

Lastly, the volume of procedures performed by a dedicated service ensures that each clinician performs a sufficient volume to maintain expertise and maximize patient safety. Future work could focus on investigating issues of patient satisfaction because the patient experience is one of four key domains in the CMS Value-Based Purchasing Program, which rewards hospitals with incentive payments for the quality of care they provide.^25^

## CONCLUSIONS

Our PA- and NP-run procedure service is well used at a large urban academic center. The demand for procedures is substantial and continues to grow every year. As structured, the core group of clinicians continues to have primary coverage responsibilities in the hospitalist service. Ongoing internal discussions continue as to whether the procedure service should be a separate service similar to the thoracentesis and PICC services at our institution; this would relieve the procedure service from any primary patient care duties. As the procedure service has evolved, the clinicians have reduced their patient panels, but there is a reluctance for more PAs and NPs to become trained in procedures because of the perception of an increased burden of work in an already full day.

Additionally, efforts continue to explore ways to perform procedures safely overnight. One strategy is to recruit more PAs and NPs to extend procedure service coverage to overnight. Additionally, the procedure service could be used to develop curriculum for trainees to understand the indications for bedside procedures as well as provide more direct supervision to trainees. In this way, residents could gain proficiency to enhance safety during nights, when they may need to perform procedures in the absence of the support of the procedure service.^5^ Finally, expanding the menu of procedures performed by the procedure service would benefit patients and expedite hospital-based care. Procedures such as joint aspirations and nerve blocks have been discussed.

We have demonstrated that a PA- and NP-run procedure service can provide safe and high-quality care. The steadily increasing volume of requests shows that the procedure service is well accepted across all hospital service lines, even in the absence of attending physician involvement. This may provide a model for creating a procedure service at other institutions similar to ours.
